# Prognostic impact of gastrointestinal bleeding and expression of PTEN and Ki-67 on primary gastrointestinal stromal tumors

**DOI:** 10.1186/1477-7819-12-89

**Published:** 2014-04-09

**Authors:** Hao Wang, Ping Chen, Xin-Xin Liu, Wei Zhao, Lei Shi, Xue-Wen Gu, Chang-Ren Zhu, Hai-Hang Zhu, Liang Zong

**Affiliations:** 1 Department of Gastrointestinal Surgery, Clinical Medical College of Yangzhou University, No. 98, Nan-Tong West Road, Yangzhou, Jiangsu 225001, P.R. China; 2 Department of Pathology, Clinical Medical College of Yangzhou University, Yangzhou, Jiangsu Province 225001, P.R. China; 3 Department of Gastroenterology, Clinical Medical College of Yangzhou University, Yangzhou, Jiangsu Province 225001, P.R. China; 4 Department of Gastrointestinal Surgery, Graduate School of Medicine, University of Tokyo, 7-3-1 Hongo, Bunkyo-ku, Tokyo 113-8655, Japan

**Keywords:** gastrointestinal bleeding, gastrointestinal stromal tumors, immunohistochemistry, Ki-67 labeling index, prognosis, PTEN, tissue microarray

## Abstract

**Background:**

Prognostic indicators for gastrointestinal stromal tumors (GISTs) are under investigation. The latest risk classification criteria may still have room for improvement. This study aims to investigate prognostic factors for primary GISTs from three aspects, including clinicopathological parameters, immunohistochemical (IHC) expression of PTEN, and Ki-67 labeling index (LI), and attempts to find valuable predictors for the malignancy potential of primary GISTs.

**Methods:**

Tumor samples and clinicopathological data from 84 patients with primary GISTs after R0 resection were obtained. Immunohistochemical analysis was performed based on tissue microarray (TMA) to estimate expression of PTEN and Ki-67 in tumor cells.

**Results:**

The cut-off point of Ki-67 LI was determined as 1%, using a receiver operator characteristic test with a sensitivity of 71.7% and a specificity of 64.5%. Univariate analysis demonstrated the following factors as poor prognostic indicators for relapse-free survival (RFS) against a median follow-up of 40.25 months: gastrointestinal (GI) bleeding (*P* = 0.009), non-gastric tumor location (*P* = 0.001), large tumor size (*P* = 0.022), high mitotic index (*P* < 0.001), high cellularity (*P* = 0.012), tumor rupture (*P* = 0.013), absent or low expression of PTEN (*P* = 0.036), and Ki-67 LI >1% (*P* = 0.043). Gastrointestinal bleeding (hazard ratio, 3.85; 95% confidence interval, 1.63 to 9.10; *P* = 0.002) was a negative independent risk predictor in multivariate analysis, in addition to tumor size (*P* = 0.023), and mitotic index (*P* = 0.002). In addition, GI bleeding showed a good ability to predict recurrence potential, when included in our re-modified risk stratification criteria.

**Conclusions:**

This study suggests that GI bleeding is an independent predictor of poor prognosis for RFS in primary GISTs. Expression of PTEN and Ki-67 are correlated with high risk potential and may predict early recurrence in univariate analysis.

## Background

Gastrointestinal stromal tumors (GISTs) constitute the most common mesenchymal neoplasms of the gastrointestinal (GI) tract, and the incidence has increased significantly over the past three decades [1,2]. Gain-of-function mutations in *c-kit* and, less commonly, the platelet derived growth factor receptor α (*PDGFRA*) oncogene are believed to be the driving force of GISTs [3,4]. All GISTs have malignant potential, varying from small lesions to aggressive sarcomas. Disease relapse is not uncommon, even where tumors are R0 resected. The application of imatinib mesylate (IM), a small-molecule tyrosine kinase inhibitor, has dramatically promoted the disease-free survival of GISTs. However, side effects and resistance to IM pose new challenges in the management of GISTs. Thus, an accurate risk classification scheme has become increasingly crucial for selecting patients who are most likely to benefit from adjuvant IM therapy. National Institutes of Health (NIH) consensus criteria [5], Armed Forces Institute of Pathology (AFIP) criteria [6], and modified NIH consensus criteria [7] are used frequently to estimate the risk of recurrence after surgery in GISTs. However, even the latest risk stratification system may still have room for improvement.

Most GISTs initially manifest as GI bleeding. We have noted in clinical practice that patients presenting with GI bleeding have appeared to fare worse than their counterparts. Few, if any, studies have addressed the prognostic implication of this important manifestation and its correlation with risk classification.

The *PTEN* (phosphatase and tensin homolog deleted from chromosome 10) gene is considered ‘the most highly mutated tumor-suppressor gene in the post-p53 era’ [8]. By dephosphorylating phosphoinositol 3,4,5-triphosphate (PIP3), *PTEN* negatively controls the activation of the PI3-kinase/Akt pathway, which has been found to be a crucial survival cell signaling transduction in GISTs [9], and thus functions as a proapoptosis factor. Also, *PTEN* plays a role in the control of the cell cycle and cell migration [10,11]. Loss of PTEN protein expression or function has been reported in many human cancers, including ovarian, endometrial, and prostate carcinoma, and breast cancer [12].

Ki-67, a nuclear protein universally expressed among proliferating cells and absent in quiescent cells [13], is one of the most frequent biomarkers investigated on GISTs. Although previous studies have shown that Ki-67 labeling index (LI) could be used in predicting the malignant potential of GISTs [14], conflicting opinions have challenged whether its expression level provides better prognostic utility than mitotic rate [15].

By using tissue microarray (TMA)-based immunohistochemistry (IHC), we aimed to analyze the impact of aforementioned factors, including clinicopathological parameters, PTEN expression, and Ki-67 LI, on the recurrence risk of R0 resected primary GISTs to identify potential new indicators that might better predict their clinical behavior and prognosis.

## Methods

### Patients and specimens

In total, 175 GIST cases, diagnosed and treated between January 2005 and January 2011, were retrieved from the hospitalization archives of the clinical medical college of Yangzhou University/Northern Jiangsu People’s Hospital (NJPH), Yangzhou, China. Of these patients, 133 received surgical resection. The inclusion criteria for this study were: (1) primary localized GISTs with R0 resection; (2) no other synchronous primary tumors; and (3) preoperative or postoperative adjuvant treatment (chemotherapy, radiotherapy and IM) were not given. Of these, 49 patients were excluded owing to: non-R0 resection (*n* = 7), receiving adjuvant treatment (*n* = 15), having other synchronous tumors (*n* = 13), missing clinical data (*n* = 8) or inadequate material for histological examination (*n* = 6). Thus, 84 GIST patients with full clinicopathological records and adequate formalin-fixed paraffin-embedded (FFPE) tissue blocks were enrolled in the current study. The FFPE tumor specimens were obtained from the archives of the Department of Pathology at NJPH.

### Follow-up

Follow-up information was obtained from medical charts, the hospital tumor registry, or direct contact with patients or their family. Recurrence or metastasis was considered the most suitable event for survival analysis because overall survival could be biased by the introduction of IM as a treatment for recurrent and metastatic GISTs. Relapse-free survival (RFS) was calculated from the date of surgical resection to the date of GIST recurrence or metastasis or to the last follow-up date, if GIST without recurrence or metastasis.

### Clinicopathological parameters

All GISTs were initially diagnosed as GI mesenchymal (non-epithelial) tumors by H & E staining, and further confirmed by positive IHC staining of CD117 or DOG-1, with or without CD 34, desmin, SMA, or S-100 positive expression. If the specimen was negative for both CD 117 and DOG-1, DNA mutation analysis of *c-kit* gene exons 9, 11, 13, and 17 or *PDGFRA* gene exons 12 and 18 were employed. Clinical data including age, sex, initial manifestation, primary tumor site, tumor size, and tumor rupture (before or during surgery) were obtained from medical records. Gastrointestinal bleeding was identified by the presence of hematemesis, black stools, or positive fecal occult blood, with or without anemia. Tumor size (maximum diameter) was measured by pathologists before the specimen was fixed. Histopathological parameters for analysis were as follows: predominant cell type (spindle, epithelioid, or mixed), mitotic index (per 50 randomly selected high power fields), tumor necrosis, cellularity (paucicellular (≤25% cells, >75% stroma), moderate (cells vs. stroma between 25% and 75%), or high (≥75% cells, <25% stroma)). Risk stratification was made based on modified NIH consensus criteria encompassing four factors: size, mitotic index, site, and rupture [7] (Table 1).

**Table 1:** **Modified NIH consensus criteria for GISTs risk stratification (adapted from**[7]**)**

**Risk category**	**Tumor size (cm)**	**Mitotic index (per 50 high power fields)**	**Primary tumor site**
Very low risk	<2.0	≤5	Any
Low risk	2.1 to 5.0	≤5	Any
Intermediate risk	2.1 to 5.0	>5	Gastric
	<5.0	6-10	Any
	5.1 to 10.0	≤5	Gastric
High risk	Any	Any	Tumor rupture
	>10 cm	Any	Any
	Any	>10	Any
	>5.0	>5	Any
	2.1 to 5.0	>5	Non-gastric
	5.1 to 10.0	≤5	Non-gastric

### Tissue microarray construction

The FFPE tissue blocks and corresponding H & E stained slides were used for TMA sampling. Two trained pathologists (XWG and CRZ) reviewed all the H & E stained slides and selected the most representative areas of tumor cells for tumor sampling. An MTA-1 manual tissue arrayer (Beecher Instruments, Sun Prairie, WI, USA) was used to punch 2.0-mm-diameter cylinders from each donor block and transfer them to the recipient paraffin block. Four-μm-thick multiple sections were cut from the TMA using a Leica RM2165 fully motorized rotary microtome (Leica Instruments GmbH, Nussloch, Germany), and prepared for subsequent IHC staining.

### Immunohistochemistry staining

The TMA slides were deparaffinized with xylene and dehydrated through a series of alcohol solutions. Endogenous peroxidase was quenched with 0.3% hydrogen for 10 min at room temperature. Antigen retrieval was performed with heat-induced methods using EDTA buffer (pH 8.0) for 15 min at 95°C, then cooled at room temperature for 20 min and washed with PBS. After being blocked with 5% fetal calf serum for 20 min at room temperature, sections were incubated with primary antibodies in an appropriate dilution overnight at 4°C. The primary antibodies used in the study were PTEN (1:50; clone D4.3; #9188P; Rabbit monoclonal antibody immunoglobulin G (IgG); Cell Signaling Technology, Danvers, MA, USA), which recognizes endogenous levels of total PTEN protein, and Ki-67 (1:200; clone D2H10; #9027S; Rabbit monoclonal antibody IgG; Cell Signaling Technology, Danvers, MA, USA), which detects endogenous levels of total Ki-67 protein. After washing three times in PBS, primary antibodies were detected using a horseradish peroxidase-labeled polymer (PowerVision Poly-HRP anti-Rabbit IgG; Leica Microsystems, Buffalo Grove, IL, USA) for 30 min at room temperature. Then 3,3′-diaminobenzedine (DAB) was used to visualize the antigens. Finally, slides were counterstained with hematoxylin, dehydrated, and mounted. As negative controls, the primary antibodies were replaced with PBS. The known positive tissue sections served as positive internal controls.

### Immunohistochemistry assessment

All TMA spots were scanned at a high resolution (20×) using the Aperio system (Aperio Technologies, Inc., Vista, CA, USA) and scored manually on computer screen independently by two pathologists (XWG and CRZ) in a blinded manner. The scoring of PTEN expression was based on the intensity and extent of staining and was evaluated according to the following histological scoring method. The mean proportion of staining cells was determined semiquantitatively and scored as follows: 0 for staining <1%, 1 for 1 to 25%, 2 for 26 to 50%, 3 for 51 to 75%, and 4 for >75% of the examined cells. Staining intensity was graded as follows: 0, negative staining; 1, weak staining; 2, moderate staining; 3, strong staining. The histological score (H-score) for each specimen was computed by the formula:

H-score = proportion score × intensity score

A total score of 0 to 12 was calculated and graded as negative (−, score: 0), weak (+, score: 1 to 4), moderate (++, score: 5 to 8) or strong (+++, score: 9 to 12). The Ki-67 LI was estimated as the percentage of Ki-67 positive cell nuclei by counting 500 to 1000 cells in the region of the tumor with the greatest density. In cases where the score difference was ≥2 for H-score and 10% for LI, the slides were re-examined and a consensus was reached by the observers. The mean score from each individual was calculated.

### Statistical analysis

All statistical analyses were calculated using IBM SPSS Statistics, version 19 (IBM, New York, USA). The IHC scores from each observer were compared for interobserver reliability using a two-way random effect model with absolute agreement definition. The average measure intraclass correlation coefficient (ICC) was obtained from these results. The Pearson’s χ-square and Fisher’s exact test were applied to examine the association between biomarker expression and clinicopathological variables. Spearman’s correlation coefficient was also calculated when needed. A receiver operator characteristic (ROC) was used to determine the best Ki-67 LI cut-off point. Univariate analyses used the Kaplan-Meier estimates, and were compared by the log-rank test. The evaluation of independent factors for RFS was carried out with the Cox proportional hazards model. *P* < 0.05 was indicative of statistical significance.

### Ethics

The Regional Ethical Committees of the NJPH approved the study. Written informed consent was obtained from all patients.

## Results

### Clinicopathological variables

The median age for the study cohort of 84 patients, 46 men and 38 women, was 61.5 years (range, 23 to 78 years), with 43 patients (51.2%) aged ≥60 years. Primary manifestations of GISTs were as follows: abdominal distention or pain (*n* = 36), GI bleeding (*n* = 24), obstruction (*n* = 6), tumor perforation or rupture (*n* = 3), weight loss (*n* = 2), and incidental detection during imaging procedures or endoscopic screening (*n* = 13). The GISTs were located in the stomach (*n* = 56), small intestine (*n* = 20), colorectum (*n* = 5), and intraperitoneum with unknown primary origin (*n* = 3). Resection was performed by open laparotomy, except in 15 patients who underwent laparoscopic resection. The tumor size varied from 1.5 to 20 cm (median, 5.3 cm). Histologically, the spindle cell type was most common (*n* = 63), followed by epithelioid cell type (*n* = 13) and mixed type (*n* = 8). The mitotic index, necrosis, and more detailed clinicopathological variables are summarized in Table 2.

**Table 2:** Clinicopathological characteristics in 84 patients with primary GISTs

**Characteristic**	**Number (%)**
**Sex**	
Male	46 (54.8)
Female	38 (45.2)
**Age (years)**	
≤60	41 (48.8)
>60	43 (51.2)
Range (median)	23 to 78 (61.5)
**Clinical manifestation**	
Abdominal discomfort or pain	36 (42.9)
Gastrointestinal bleeding	24 (28.6)
Obstruction	6 (7.2)
Perforation or rupture	3 (3.6)
Weight loss	2 (2.4)
Asymptomatic	13 (15.5)
**Primary tumor site**	
Stomach	56 (66.7)
Small intestine	20 (23.8)
Colorectum	5 (6.0)
Intraperitoneally with unknown origin	3 (3.6)
**Tumor size (cm)**	
≤5	41 (48.8)
5.1 to 10	34 (40.5)
>10	9 (10.7)
Range (median)	1.5 to 20 (5.3)
**Predominant cell type**	
Spindle	63 (75.0)
Epithelioid	13 (15.5)
Mixed	8 (9.5)
**Mitotic index (per 50 high power fields)**	
0 to 5	53 (63.1)
5.1 to 10	21 (25.0)
>10	10 (11.9)
**Cellularity**	
Paucicellular	11 (13.1)
Moderate	33 (39.3)
High	40 (47.6)
**Necrosis**	
Yes	34 (40.5)
No	50 (59.5)
**Tumor rupture**	
Yes	5 (6.0)
No	79 (94.0)
**Risk classification**	
Very low	5 (6.0)
Low	26 (31.0)
Moderate	17 (20.2)
High	36 (42.9)
**Follow-up**	
Relapse-free survival	59 (70.2)
Recurrent	16 (19.1)
Metastasis	9 (10.7)

### Interobserver variability

Interobserver scoring agreement was tested for the two biomarkers. The average measure ICCs were 0.93 for PTEN (95% confidence interval (CI), 0.89 to 0.96; *P* < 0.001) and 0.83 for Ki-67 LI (95% CI, 0.73-0.89; *P* < 0.001).

### PTEN expression

**Figure 1:**
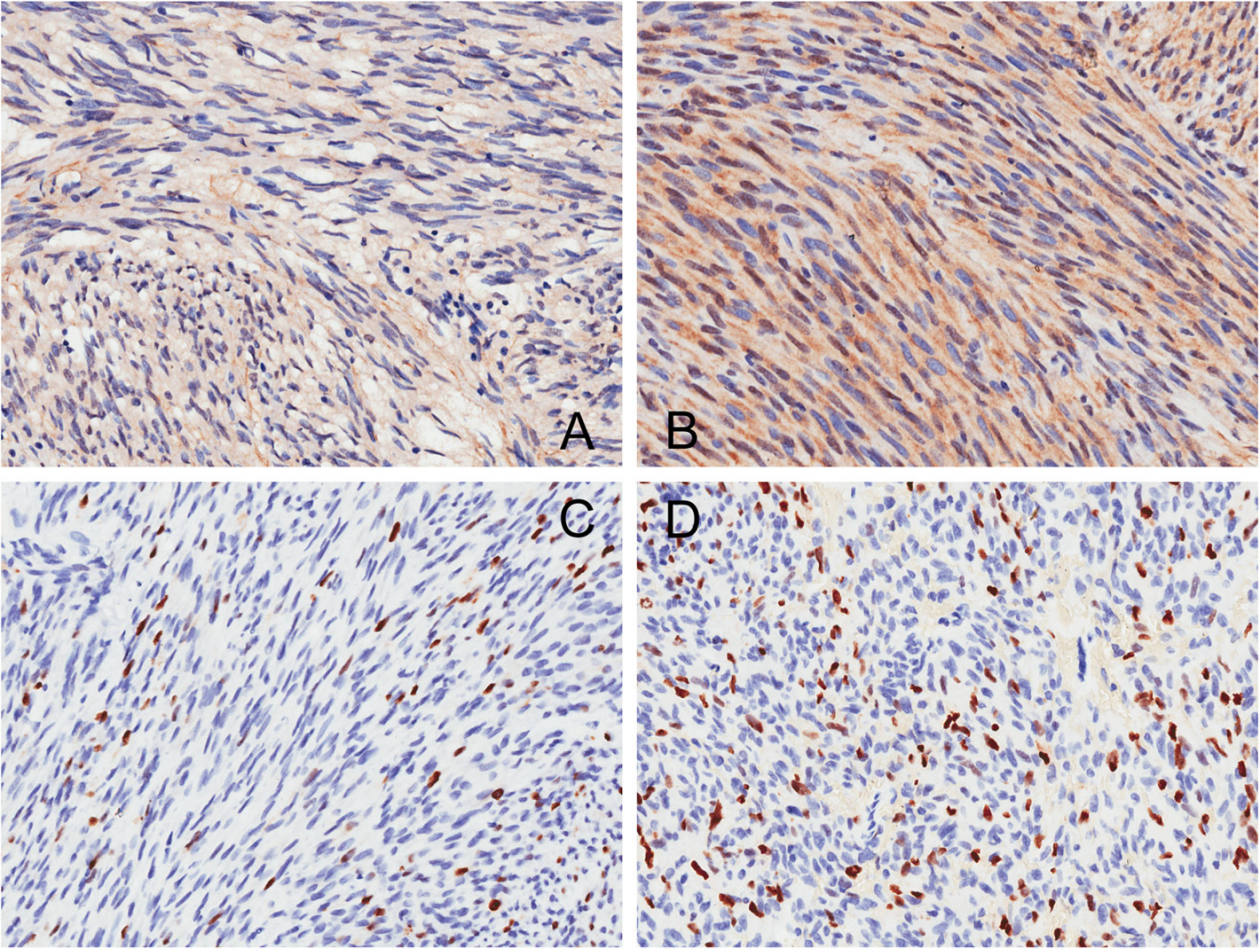
**IHC analysis of GISTs representing different expression of PTEN and Ki-67 (3,3′-Diaminobenzidine, magnification × 20). (A)** PTEN, cytoplasmic staining (H-score, 4) and **(B)** both cytoplasmic and nuclei staining (H-score, 8). **(C)** Ki-67 nuclei staining in 5% and **(D)** 20% of tumor cells.

The PTEN showed expression in the cytoplasm or in both the cytoplasm and the nuclei of tumor cells in the majority of cases, while pure nuclei staining was not observed. According to the H-score scheme, PTEN expression was considered as (−) in 11 patients (13.1%), (+) in 33 patients (39.3%), (++) in 37 patients (44.0%), and (+++) in 3 patients (3.6%) (Figure 1A,B). There was a significant association between PTEN expression and risk stratification (*P* = 0.001), as indicated by Fisher’s exact test. Furthermore, Spearman’s correlation test revealed that PTEN expression was inversely correlated with risk stratification (*R*_s_ = −0.318, *P* = 0.003) (Table 3).

**Table 3:** Correlation between the expression of PTEN, Ki-67 and risk grade in 84 GISTs

	**Risk classification number (%)**				***P***	**Spearman’s correlation**	
	**Very low**	**Low**	**Intermediate**	**High**		***R***_**s**_	***P***
**PTEN**					0.001*	−0.318	0.003
(−)	2 (2.4)	3 (3.6)	1 (1.2)	5 (6.0)			
(+)	–	4 (4.8)	7 (8.3)	22 (26.2)			
(++)	3 (3.6)	17 (20.2)	8 (9.5)	9 (10.7)			
(+++)	–	2 (2.4)	1 (1.2)	–			
**Ki-67 labeling index**					<0.001*	0.434	<0.001
≤1%	5 (6.0)	15 (17.9)	8 (9.5)	7 (8.3)			
>1%	–	11 (13.1)	9 (10.7)	29 (34.5)			

*Fisher’s exact test.

### Ki-67 LI

Ki-67 LI ranged from 0% to 23.4% (median, 1.1%) (Figure 1C,D). The threshold value of Ki-67 LI was determined as 1% by ROC using relapse as basis, with a sensitivity of 71.7% and specificity of 64.5% (area under the curve, 0.651; 95% CI, 0.530 to 0.772; *P* = 0.021). Thirty-five cases (41.7%) showed Ki-67 LI ≤1%, and 49 (58.3%) showed Ki-67 LI >1%. There was a significant association between Ki-67 LI >1% and risk stratification, as indicated by Fisher’s exact test (*P* < 0.001) and Spearman’s correlation test (*R*_s_ = 0.434, *P* < 0.001) (Table 3).

### Follow-up

Follow-up data were available for all patients. The median follow-up time was 40.3 months (range, 4 to 97 months) for patients free of recurrence or metastasis, and the cumulative 1, 3, and 5-year rates for RFS were 98.8%, 79.8%, and 70.2%, respectively. Twenty-five patients experienced tumor relapse during the follow-up period, including local recurrence in the abdominopelvic cavity (*n* = 16) and metastases to liver (*n* = 9). Metastasis to the lymph nodes was not observed.

### Univariate survival analysis

**Figure 2:**
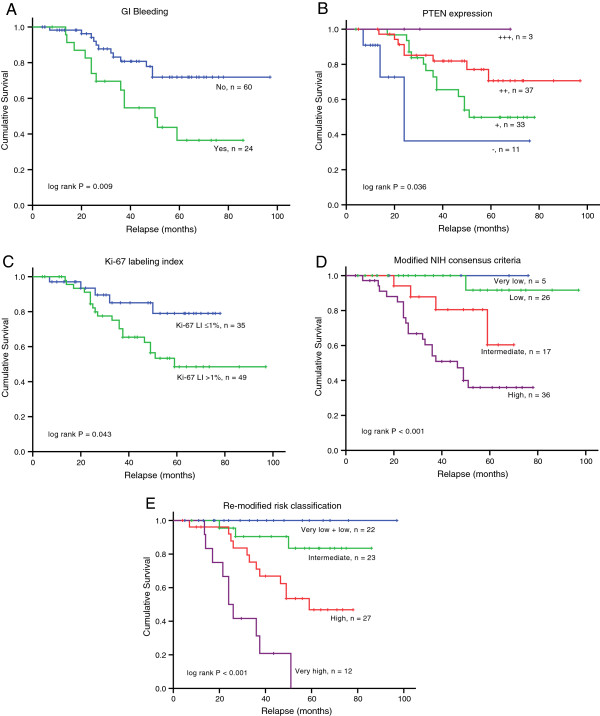
**Relapse-free survival analysis of 84 patients with primary GISTs.** Kaplan-Meier curve analysis demonstrated a worse relapse-free survival for patients presenting with **(A)** gastrointestinal (GI) bleeding, **(B)** absent or low PTEN expression, and **(C)** Ki-67 > 1%. By including GI bleeding as an independent prognostic indicator, our re-modified risk stratification system **(E)** showed a better ability to predict recurrence in primary GISTs than modified NIH consensus criteria **(D)**.

Univariate survival analysis demonstrated that GI bleeding (*P* = 0.009), primary site (*P* = 0.001), tumor size (*P* = 0.022), mitotic index (*P* < 0.001), cellularity (*P* = 0.012), tumor rupture (*P* = 0.013), modified NIH risk stratification (*P* < 0.001), PTEN expression pattern (*P* = 0.036), and Ki-67 LI (*P* = 0.043) were all significant prognostic parameters for RFS. Correlations of clinicopathological and immunohistochemical factors to RFS are shown in Table 4 and Figure 2.

**Table 4:** Univariate analysis of factors influencing RFS

**Factors**		**5-year RFS rate (%)**	***P***
Sex	Male: female	59.6:59.5	0.702
Age (years)	≤60: >60	54.6:64.3	0.306
Gastrointestinal bleeding	Yes: no	36.4:71.9	0.009
Primary tumor site	Gastric: non-gastric	73.1:36.0	0.001
Tumor size (cm)	≤5: 5.1 to 10: >10	79.9:47.0:33.3	0.022
Predominant cell type	Spindle: epithelioid: mixed	63.0:45.0:51.4	0.423
Mitotic index (per 50 high power fields)	0 to 5: 5.1 to 10: >10	74.9:46.1:22.2	<0.001
Cellularity	Paucicellular or moderate: high	69.6: 50.2	0.012
Necrosis	Yes: no	48.9: 69.2	0.09
Tumor rupture	Yes: no	20.0: 63.3	0.013
Risk classification	Very low: low: intermediate: high	100.0:91.7:60.4:36.0	<0.001
PTEN	(−): (+): (++): (+++)	36.4:49.8:70.7:100.0	0.036
Ki-67 labeling index	≤1%: >1%	79.0:48.5	0.043

PTEN, Phosphatase and tensin homolog deleted from chromosome 10*.*

### Multivariate survival analysis

Only those factors showing a significant correlation with RFS in the univariate survival analysis were selected for inclusion in the Cox proportional hazards model in stepwise forward and backward selection strategies. The modified NIH risk stratification was excluded from the multivariate analysis because it was based on tumor site, size, mitotic index, and rupture, and markedly correlated with each of these parameters. The results of the Cox regression analysis are listed in Table 5. Gastrointestinal bleeding (hazard ratio, 3.85; 95% CI, 1.63 to 9.10; *P* = 0.002), larger tumor size (*P* = 0.023), and high mitotic index (*P* = 0.002) were statistically significant independent negative prognostic indicators for RFS.

**Table 5:** Results of Cox regression analysis (stepwise forward method) summarizing significant independent prognostic factors for RFS

**Factor**	**Hazard ratio**	**95% confidence interval**	***P***
**Gastrointestinal bleeding**			
No	1.00		
Yes	3.85	1.63 to 9.10	0.002
**Tumor size**			0.023*
≤5 cm	1.00		
5.1 to 10 cm	1.86	0.67 to 5.15	0.232
>10 cm	6.71	1.70 to 26.51	0.007
**Mitotic index**			0.002*
0 to 5 (per 50 high power fields)	1.00		
5.1 to 10 (per 50 high power fields)	5.72	2.09 to 15.64	0.001
>10 (per 50 high power fields)	3.44	1.13 to 10.45	0.029

*Overall significance as a prognostic factor.

### Proposal for re-modifying GIST risk stratification system

Based on these results, we consider that the current NIH consensus criteria could be further refined and expanded by including GI bleeding as an important independent prognostic indicator. For example, a patient from the ‘low-risk’ group according to the modified NIH consensus criteria who presented with GI bleeding preoperatively would be upgraded in risk to the re-modified ‘intermediate risk’ group. By such analogy, the risk classification could be reclassified into five groups: ‘very low’, ‘low’, ‘intermediate’, ‘high’, and ‘very high’. As shown in Kaplan-Meier curves, this re-modified risk stratification system demonstrated a better ability to predict prognosis of primary resectable GISTs (*P* < 0.001) (Figure 2D,E).

## Discussion

In this retrospective study based on follow-up data, we have assessed the impact of a set of clinicopathological parameters and the role of PTEN and Ki-67 on the prognostic of primary GIST patients treated with surgery alone. Our main findings were as follows: (1) GI bleeding, a common manifestation for primary GISTs, was an independent predictor of worse prognosis for RFS; (2) the IHC underexpression of PTEN was associated with worse RFS at univariate analysis; (3) a Ki-67 LI >1% was associated with worse RFS at univariate analysis; (4) GI bleeding could be considered an additional prognostic indicator in the GIST risk classification scheme.

Gastrointestinal bleeding is one of the most common clinical symptoms of GIST [16]. In a long-term follow-up of 1,765 cases of gastric GISTs [17], 54.5% patients presented with GI bleeding, ranging from chronic insidious hemorrhage with or without anemia to acute melena or hematemesis. Although we could not reproduce this high incidence, our data still illustrate an incidence of GI bleeding as high as 28.6%. Ulceration and mucosal invasion are the most common causes. Ulceration of the mucosa is commonly manifest in GISTs (39.6%), and is a statistically significant predictor for progressive disease (*P* < 0.0003) [16]. Although mucosal invasion is rare, it has been shown in a surprisingly high proportion of progressive GISTs (22 of 24 cases) [18]. Research regarding the prognostic implication of GI bleeding on RFS in GISTs is scant; in addition to one study [19] that found a statistical significant prediction value on univariate analysis but not on multivariate analysis. Our data demonstrated that GI bleeding was an independent predictor of poor prognosis for RFS. As shown in Figure 2E, the curves of the ‘very low’ and ‘low’ groups merged totally, which might suggest that these two groups in our proposal risk classification system can be incorporated into the same group. In that case, there would still be four groups, while the ‘very high’ group highlights very well that GI bleeding is a negative prognostic indicator. Moreover, Kaplan-Meier curves illustrated that patients whose risk category upgraded due to GI bleeding in our re-modified risk stratification scheme are more likely to suffer from disease relapse, suggesting that GI bleeding, a clinical feature worth recording, might be added to the risk classification system in further studies.

Original researches performed in murine models, suggesting that PTEN might be involved in the aberrant activation of cell signaling transduction in GISTs tumorigenesis; however, the results are inconsistent. By using a *Kit*^K641E^ knock-in mouse model of GIST, Deneubourg *et al.*[20] indicated that the upregulation of PTEN might act as a negative feedback mechanism to limit PI3-kinase activation downstream of Kit in a context of oncogenic mutation. On the other hand, in a panel of GIST xenograft mouse models, Floris and colleagues [21] illustrated that homozygous PTEN loss was identified in the GIST-PSW (GIST biopsy tumor cell with KIT exon 11 mutation) and GIST882Ly (GIST cell line harboring homozygous KIT exon 13 mutation) xenografts, while heterozygous PTEN loss was observed in a GIST48 (GIST cell line carrying both KIT exon 11 and 17 mutation) xenograft. Importantly, they also confirmed the lack of PTEN protein expression in the GIST-PSW and GIST882Ly xenografts. PTEN protein expression has previously been investigated in clinical GISTs tissue samples by IHC, and a clear relationship between low or absent PTEN expression and poor prognosis was revealed, either alone or in combination with other biomarkers [22,23]. Our study, adopting a PTEN IHC H-score method, provides further support for the role of absent or low expression (H-score, 0 to 4) of PTEN (in 52.4% GISTs) as a predictor of recurrence in patients with primary GIST, although the result was not statistically significant on multivariate analysis.

Additionally, diffuse cytoplasmic or both cytoplasmic and nucleic PTEN immunostaining was observed, while pure nuclei staining was not observed in this study. However, exclusively nuclear PTEN immunoreactivity in GIST was previously reported in two studies [22,23]. A possible reason for this discrepancy could be the different anti-PTEN antibodies used in IHC analysis. Our findings are consistent with Deneubourg and his colleagues, who revealed PTEN subcellular expression in the cytoplasm and partly in the nucleus by immunofluorescence in human GIST882 cells [20]. PTEN has been shown to shuttle between the cytoplasm and nucleus. Its different subcellular localization might imply different molecular functions. It is worth noting that negative nuclear PTEN immunostaining, unlike cytoplasmic PTEN expression, is an independent prognostic factor for survival in esophageal squamous cell carcinoma [24]. Accordingly, further studies are warranted, to define the prognostic implication of different subcellular PTEN expression in GISTs.

Although Ki-67 shows the most potential as a prognostic indicator for GISTs in the literature, the exact threshold value of Ki-67 LI for malignant predicting remains debatable. In documented research, different cut-off points have been suggested: 0.82% [25], 2.7% [26], 3% [27], 5% [15,23], 6% [28], or 10% [29]; however few of these cut-off points were established based on ROC analysis. In our series, a 1% LI was confirmed as the best threshold value for relapse in ROC analysis, with 71.7% sensitivity and 64.5% specificity. Whether Ki-67 LI could replace mitotic counting in prognostication remains controversial [30]. Some scholars hold that the mitotic index reflects the M stage of mitosis only; but, because Ki-67 LI can be used to recognize most of the proliferating cells in stages G1, S, and G2, it is considered to be more appropriate as an objective index of the malignancy of GISTs [31]. Molenaar *et al.*[32] also demonstrated higher observer reliability of Ki-67 than mitotic counting in assessment of mitotic activity. On the contrary, Wong *et al.*[15] advocated Ki67 LI to be inferior to mitotic index as a prognostic marker. Our study shows that a Ki-67 LI level higher than 1% is associated with a higher relapse potential of primary GISTs, on univariate survival analysis but not on multivariate analysis, and that mitotic index remains a stable prognostic indicator, on both univariate and multivariate analysis. We propose that if the mitotic index is based on counting, fewer than 50 high power fields (that is in a small biopsy sample), Ki-67 immunohistochemical analysis could be an alternative for mitotic index.

The NIH consensus criteria [5], AFIP criteria [6], and modified NIH consensus criteria [7] are frequently used to estimate the risk of recurrence after surgery in GIST, and their prognostic accuracy is generally similar [33]. NIH consensus criteria stratify risk based on tumor size and mitotic counting, whereas AFIP criteria also include tumor site. Modified NIH criteria encompass four factors: tumor size, mitotic index, site, and tumor rupture, which have been well established as important independent prognostic factors for GIST recurrence [34,35] (Table 1). Our data regarding these parameters reach a general agreement with prior reports, showing that larger tumor size and high mitotic index are steadily indicative of poor RFS for primary GISTs, in addition to tumor site and rupture, which were not identified as independent prognostic factors in multivariate analysis. In a pooled analysis of ten population-based series including 2,459 GIST patients treated with surgery alone [33], the estimated 5-year and 15-year RFS rates were 70.5% and 59.9%, respectively. In line with this study, the overall outcome after surgery in our cohort was presented as 1, 3, and 5-year RFS rates (98.8%, 79.8%, and 70.2%, respectively).

There are certain limitations in our study. First, this is a retrospective study with a limited sample size of study population and a large proportion of censored cases. Secondly, the study was conducted in one single tertiary hospital. Thus, a larger scale, multicenter prospective study with longer-term follow-up investigation is warranted. Finally, the inability of TMA approach to detect all information of tumor samples might cause bias [36]. Despite these caveats, it appears that our findings can contribute to the knowledge of prognostic biomarkers in patients with primary GISTs who are treated with surgery alone.

## Conclusions

The current NIH consensus criteria remain a stable and practical predicting tool in primary GISTs risk classification. We found that GI bleeding was an independent prognostic indicator for poor RFS in patients with primary GISTs treated with surgery alone. We proposed that the current NIH consensus criteria could be improved by including this important clinical manifestation. Whether absent or low expression of PTEN, and Ki-67 LI higher than 1% could add independently to the prognostication of GISTs should be further investigated in a larger series.

## Abbreviations

AFIP: Armed Forces Institute of Pathology; CI: confidence interval; DAB: 3,3′-diaminobenzidine; EDTA: ethylenediaminetetraacetic acid; FFPE: formalin-fixed paraffin-embedded; GI: gastrointestinal; GIST: gastrointestinal stromal tumor; H & E: hematoxylin and eosin; ICC: interclass correlation coefficient; IgG: immunoglobulin G; IHC: immunohistochemistry; IM: imatinib mesylate; LI: labeling index; NIH: National Institutes of Health; NJPH: Northern Jiangsu People’s Hospital; PBS: phosphate-buffered saline; PDGFRA: platelet derived growth factor receptor α; PIP3: phosphoinositol 3,4,5-triphosphate; PTEN: phosphatase and tensin homolog deleted from chromosome 10; RFS: relapse-free survival; ROC: receiver operator characteristic; TMA: tissue microarray.

## Competing interests

The authors declare that they have no competing interests.

## Authors’ contributions

HW, PC, WZ, XXL, LS, XWG, CRZ and LZ participated in the conception and design of the study. HW, PC and LS collected clinical information. HW, XWG and CRZ reviewed all the histological diagnoses, histological grading, selected and marked the slides for TMA construction. HW, XXL and LS performed the experiments. HW performed the statistical analysis. WZ, LS and HHZ contributed reagents, materials, and analysis tools. HW and PC drafted the manuscript. All authors read and approved the final manuscript.
